# Supplementary material for: Developing an evaluation framework for public health environmental surveillance: Protocol for an international, multidisciplinary Delphi consensus study
Source: PLoS One. 2025 May 27;20(5):e0310342. doi: 10.1371/journal.pone.0310342 (PMC12111604; doi:10.1371/journal.pone.0310342)
Supplement: S1 Table — (PDF) [file pone.0310342.s001.pdf]

**Name**	**Primary Affiliation**	**Discipline**	**Country of Residence**
Douglas Manuel	Ottawa Hospital Research Institute	Public health, infectious disease, epidemiology; Mathematical sciences	Canada
David Buckeridge	McGill University	Public health, infectious disease, epidemiology; Mathematical sciences	Canada
David Moher	Ottawa Hospital Research Institute	Public health, infectious disease, epidemiology; Communication, knowledge translation and exchange	Canada
Beate Sander	University of Toronto	Mathematical sciences; Public health, infectious disease, epidemiology	Canada
Sarah Funnell	Queen's University	Public health, infectious disease, epidemiology; Social sciences	Canada
Yoni Freedhoff	N/A	Engaged public	Canada
Jeremy Veillard	World Bank	Public health, infectious disease, epidemiology; Knowledge user	United States of America
Amy Kirby	Centers for Disease Control and Prevention	Public health, infectious disease, epidemiology; Environmental and physical sciences	United States of America
Kerrigan McCarthy	National Institute for Communicable Diseases	Public health, infectious disease, epidemiology; Environmental and physical sciences	South Africa
Farah Ishtiaq	Tata Institute for Genomics and Society	Environmental and physical sciences	India

Matthew Wade	UK Health Security Agency	Environmental and physical sciences; Mathematical sciences	United Kingdom
Bernd Gawlik	European Commission, DG Joint Research Centre	Environmental and physical sciences	Germany
