# Supplementary material for: Developing an evaluation framework for public health environmental surveillance: Protocol for an international, multidisciplinary Delphi consensus study
Source: PLoS One. 2025 May 27;20(5):e0310342. doi: 10.1371/journal.pone.0310342 (PMC12111604; doi:10.1371/journal.pone.0310342)
Supplement: S2 Table — The list of specializations or related concepts is not extensive. (PDF) [file pone.0310342.s002.pdf]

Discipline group	Discipline subgroup	Definition	Specializations or related concepts
Content experts	Public health, infectious disease, epidemiology	Understanding health-related conditions, patterns, and events within populations. The science and practice of public health management, including surveillance systems	Epidemiology, infectious disease, global health, public health, population health
	Environmental and physical sciences	Understanding and managing environmental and biologic systems	Civil engineering, environmental engineering, biological engineering, chemical engineering, environmental life sciences engineering, environmental microbiology, water microbiology, microbiology, environmental health, molecular ecology, disease ecology, evolution, hydrology, water resources, evolutional biology, veterinary science, geology, sedimentary geochemistry, analytical chemistry, analytical toxicology, environmental toxicology, virology, genetics. geology, sedimentary geochemistry, analytical chemistry, analytical toxicology, environmental toxicology, water quality and treatment, environmental chemistry
	Mathematical sciences	Understanding of quantitative analysis, modelling, and data insights; not otherwise stated in other disciplines	Mathematics, applied mathematics, statistics, bioinformatics, computer science, economics, health economics, econometrics, data science, analytics
	Social sciences	Understanding of societal, cultural, ethical, and legal aspects	Sociology, demography, indigenous studies, sustainable development, international development, sustainability, law, ethics, bioethics, behavioural sciences, environmental policy and governance

	Communication, knowledge translation and exchange	Understanding and facilitating of communication and dissemination of knowledge, ensuring that the information reaches and is understood by all parties involved with or affected by wastewater-based surveillance	Journalology, journalism, science communication, knowledge synthesis, knowledge translation and exchange
Knowledge users		A professional who does not have specialized training or qualifications in wastewater-based surveillance, but who uses surveillance to inform policy and action in their workplace	Health care (medicine, nursing, or other health care profession), government, non-governmental organization administrators, business, public policy, policy and administration, civil leadership, and executives
Engaged public		A non-expert who has a general understanding or interest in wastewater-based surveillance	Citizen science, community residents
