# Supplementary material for: Developing an evaluation framework for public health environmental surveillance: Protocol for an international, multidisciplinary Delphi consensus study
Source: PLoS One. 2025 May 27;20(5):e0310342. doi: 10.1371/journal.pone.0310342 (PMC12111604; doi:10.1371/journal.pone.0310342)
Supplement: S3 Table — Note: The regional classifications are used by the World Bank for organizing economic data, development projects, and policy analysis. These classifications are not strictly geographical but also consider economic and developmental similarities and relationships. (PDF) [file pone.0310342.s003.pdf]

World region	Examples of included countries
East Asia and Pacific	China, Indonesia, Philippines, Malaysia, Australia
Europe and Central Asia	United Kingdom, Norway, Germany, France, Portugal
Latin America and the Caribbean	Mexico, Brazil, Jamaica, Haiti, Argentina
Middle East and North Africa	Morocco, Algeria, Saudi Arabia, Iran, Iraq
North America	Canada, United States, Bermuda
South Asia	India, Pakistan, Bangladesh, Nepal, Bhutan
Sub-Saharan Africa	Nigeria, South Africa, Kenya, Ethiopia, Tanzania

Source: The World Bank.
