# Supplementary material for: Developing an evaluation framework for public health environmental surveillance: Protocol for an international, multidisciplinary Delphi consensus study
Source: PLoS One. 2025 May 27;20(5):e0310342. doi: 10.1371/journal.pone.0310342 (PMC12111604; doi:10.1371/journal.pone.0310342)
Supplement: S3 Appendix — (PDF) [file pone.0310342.s006.pdf]

1. **Impact & practical utility:**
    - Attributes should advance surveillance system goals, improve population health, and inform decision-making.
    - This property emphasizes the translational aspects of surveillance, bridging data gathering to meaningful, measurable outcomes.
2. **Scientific rigor & validity:**
    - Attributes must be based on well-established, validated methods relevant to wastewater-based surveillance.
    - This ensures that collected data can be trusted for robust insights, comparisons, and the formation of evidence-based policies or interventions.
3. **Feasibility, adaptability, & resource implications:**
    - Attributes should balance ease of measurement with data quality and allow adaptation to different settings.
    - This emphasizes practicality considering limited resources and varied technical capacities and ensures applicability across sewered/unsewered and varying income and geographic settings.
4. **Equity, mitigation of bias, & inclusiveness:**
    - Attributes should actively minimize disparities in data collection, analysis, and interpretation.
    - This supports just and inclusive surveillance, particularly for low-resource settings and marginalized populations.

These four properties were synthesized from the following list of properties from previous performance evaluation frameworks from public health and health systems [1–8]. Equity is a property that was not previously identified but was added following the Expert Group discussion of potential properties and the study's goals and objectives.

Previously used properties

1. **Importance:**
    - This criterion evaluates the measure's relevance and significance in wastewater-based surveillance. High-performing surveillance systems should prioritize measures directly related to the goals and outcomes they aim to achieve.

2. **Reliability (reproducibility) of data:**
    - This criterion assesses whether the data collected using the metric is reliable. Ensuring that the metric produces consistent results when measured multiple times is essential.

3. **Responsiveness:**
    - Responsiveness assesses the ability of the measure to capture changes and variations in the surveillance system's performance. A responsive measure should be sensitive to improvements or deteriorations in the system's functioning.

4. **Sensitivity to change:**
    - Sensitivity to change assesses whether the metric can detect meaningful variations or improvements in performance over time. Metrics need to be responsive to changes in the wastewater-based surveillance system.

5. **Mutability or improvability:**
    - This criterion considers whether the measure is amenable to improvement or change. Measures that can be modified or enhanced to reflect better the evolving needs of wastewater-based surveillance are valuable.

6. **Burden of data collection:**
    - This criterion considers the practicality of data collection for the metric. It assesses whether data collection is feasible and cost-effective and whether the necessary data are readily available and accessible.

7. **Contextual validity:**
    - Contextual validity examines whether the metric remains valid and relevant in different contexts related to wastewater-based surveillance and whether its applicability extends to various surveillance settings and situations.

8. **Construct validity:**
    - Construct validity assesses whether the metric is related to other indicators measuring the same aspects of wastewater-based surveillance and whether it aligns with established constructs and measures in the field.

9. **Content validity:**
    - Content validity assesses whether the metric effectively captures the specific aspects of wastewater-based surveillance it is intended to assess. It evaluates whether the metric's content aligns with the targeted dimensions of surveillance.

10. **Timeliness and accessibility of data:**
    - Timeliness assesses how quickly data can be collected and reported using the metric in wastewater-based surveillance. Accessibility evaluates whether the data required for the metric are readily available and accessible.

11. **Ethical and legal considerations:**
    - These criteria evaluate whether the metric raises ethical or legal concerns in wastewater-based surveillance, such as privacy issues, consent, and compliance with relevant regulations.

12. **User acceptance and utility:**
    - User acceptance assesses whether the metric is user-friendly and accepted by professionals and stakeholders in wastewater-based surveillance. Utility evaluates the practical usefulness of the metric for decision-making and quality improvement.

References:

1. Locke EA, Latham GP. A theory of goal setting & task performance. Prentice-Hall, Inc; 1990.

2. Veillard J, Champagne F, Klazinga N, Kazandjian V, Arah OA, Guisset A-L. A performance assessment framework for hospitals: the WHO regional office for Europe PATH project. Int J Qual Health Care. 2005;17: 487–496. doi:10.1093/intqhc/mzi072

3. Kaplan RS, Norton DP. Balanced scorecard. Springer; 2007.

4. Mitchell R.J., Williamson A.M., O'Connor R. The development of an evaluation framework for injury surveillance systems. BMC Public Health. 2009;9: 260. doi:10.1186/1471-2458-9-260

5. Nothacker M, Stokes T, Shaw B, Lindsay P, Sipilä R, Follmann M, et al. Reporting standards for guideline-based performance measures. Implement Sci. 2016;11: 6. doi:10.1186/s13012-015-0369-z

6. Groseclose SL, German RR, Nsubuga P. Evaluating Public Health Surveillance. Principles & Practice of Public Health Surveillance. 2010. doi:10.1093/acprof:oso/9780195372922.003.0008

7. Woodward G, Manuel DG, Goel V. Developing a balanced scorecard for public health. Toronto: Institute for Clinical Evaluative Sciences; 2004.

8. Calba C, Goutard FL, Hoinville L, Hendrikx P, Lindberg A, Saegerman C, et al. Surveillance systems evaluation: a systematic review of the existing approaches. BMC Public Health. 2015;15: 448. doi:10.1186/s12889-015-1791-5
