# Supplementary material for: Developing an evaluation framework for public health environmental surveillance: Protocol for an international, multidisciplinary Delphi consensus study
Source: PLoS One. 2025 May 27;20(5):e0310342. doi: 10.1371/journal.pone.0310342 (PMC12111604; doi:10.1371/journal.pone.0310342)
Supplement: S4 Appendix — (PDF) [file pone.0310342.s007.pdf]

PHES-EF e-Delphi Panellist Screening Questionnaire

1. **Full name:**
   Please provide your full name

   [Text box]

2. **Email address:**
   Please provide an email address that you actively monitor and that you wish to be contacted at

   [Text box]

3. **Are you 18 years of age or older?**

   Yes
   No

4. **Are you proficient in English?**

   Yes
   No

5. **Do you have professional experience or a graduate degree in a discipline that contributes (or could contribute) to wastewater-based surveillance or epidemiology?**
   See table below for a non-extensive list of disciplines relating to wastewater-based surveillance:

Discipline group	Discipline subgroup	Definition	Specializations or related concepts
Content experts	Public health, infectious disease, epidemiology	Understanding health-related conditions, patterns, and events within populations. The science and practice of public health management, including surveillance systems	Epidemiology, infectious disease, global health, public health, population health
	Environmental and physical sciences	Understanding and managing environmental and biologic systems	Civil engineering, environmental engineering, biological engineering, chemical engineering, environmental life sciences engineering, environmental microbiology, water microbiology, microbiology, environmental health, molecular ecology, disease ecology, evolution, hydrology, water resources,

DRAFT

DRAFT

			evolutional biology, veterinary science, geology, sedimentary geochemistry, analytical chemistry, analytical toxicology, environmental toxicology, virology, genetics. geology, sedimentary geochemistry, analytical chemistry, analytical toxicology, environmental toxicology, water quality and treatment, environmental chemistry
	Mathematical sciences	Understanding of quantitative analysis, modelling, and data insights; not otherwise stated in other disciplines	Mathematics, applied mathematics, statistics, bioinformatics, computer science, economics, health economics, econometrics, data science, analytics
	Social sciences	Understanding of societal, cultural, ethical, and legal aspects	Sociology, demography, indigenous studies, sustainable development, international development, sustainability, law, ethics, bioethics, behavioural sciences, environmental policy and governance
	Communication, knowledge translation and exchange	Understanding and facilitating of communication and dissemination of knowledge, ensuring that the information reaches and is understood by all parties involved with or affected by wastewater-based surveillance	Journalology, journalism, science communication, knowledge synthesis, knowledge translation and exchange
Knowledge users		A professional who does not have specialized training or qualifications in wastewater-based surveillance, but who uses surveillance to inform policy and action in their workplace	Health care (medicine, nursing, or other health care profession), government, non-governmental organization administrators, business, public policy, policy and administration, civil leadership, and executives
Engaged public		A non-expert who has a general understanding or interest in wastewater-based surveillance	Citizen science, community residents

Definitions:

- Professional experience is defined as having paid employment or professional practice in a listed specialization (current or former)
- A graduate or professional degree is defined as having obtained a Master's or Doctor of Philosophy (PhD)

Yes
No

If yes, go to 5a
If no, to 6

a) **Please select your primary discipline of expertise:**
Please provide your area of specialization in the text box (examples can be found in the right column of the table provided in question 5)

- Public health, infectious disease, epidemiology
- Environmental and physical sciences
- Mathematical sciences
- Social sciences
- Communication, knowledge translation and exchange

[Text box]

b) **Do you have a graduate degree in your discipline or area of specialization?**
If yes, please provide your degree name(s) and granting institution(s)

Yes
No

[Text box]

c) **Do you have professional experience related to your stated primary discipline?**
Professional experience is defined as having paid employment or professional practice in a listed specialization (current or former)

Yes, three or more years of experience
Yes, less than three years of experience
No

d) **Have you published any wastewater-based, environmental, or public health surveillance studies in a peer-reviewed journal?**

Yes, two or more publications
Yes, less than two publications
No

DRAFT

6. **Are you a professional with no specialized training or qualifications in wastewater-based surveillance, but who uses (or may use) surveillance data to inform policy and action in your workplace?**

   Yes
   No

   If no, go to 7

7. **Are you a non-expert with relevant lived experience, a general understanding, or interest in wastewater-based surveillance?**

   Yes
   No

DRAFT
